# Analysis of urgent/emergent conversions from monitored anesthesia care to general anesthesia with airway instrumentation

**DOI:** 10.1186/s12871-021-01403-9

**Published:** 2021-06-29

**Authors:** Sang Kim, Brian A. Chang, Amreen Rahman, Hung-Mo Lin, Samuel DeMaria, Jeron Zerillo, David B. Wax

**Affiliations:** 1 Department of Anesthesiology, Critical Care & Pain Management - Hospital for Special Surgery, New York, NY 10021 USA; 2 Department of Anesthesiology, New York Presbyterian, Columbia University Irving Medical Center, New York, USA; 3 Department of Anesthesiology, Perioperative and Pain Medicine- Icahn School of Medicine at Mount Sinai, New York, USA

**Keywords:** Monitored anesthesia care, General anesthesia, BMI, Retrospective, Patient safety

## Abstract

**Background:**

Monitored Anesthesia Care (MAC) is an anesthetic service involving the titration of sedatives/analgesics to achieve varying levels of sedation while avoiding general anesthesia (GA) and airway instrumentation. The goal of our study was to determine the overall incidence of conversion from MAC to general anesthesia with airway instrumentation and elucidate reasons and risk factors for conversion.

**Methods:**

In this retrospective observational study, all non-obstetric adult patients who received MAC from July 2002 to July 2015 at Mount Sinai Hospital were electronically screened for inclusion via a clinical database. Patient, procedure, anesthetic, and practitioner data were all collected and analyzed to generate descriptive analyses. Subsequent univariate and multivariate analyses were used to identify specific risk factors associated with conversion to GA.

**Results:**

Overall, 0.50% (1097/219,061) of MAC cases were converted to GA. Approximately half of conversions were due to the patient’s “intolerance” of MAC (with or without failed regional anesthesia), while the other half were due to physiologic derangements. Body mass index, male sex, American Society of Anesthesiologists Physical Status Classification, anesthesia team composition, and surgical specialty were all associated with risk of conversion to GA.

**Conclusions:**

This is one of the first and largest retrospective studies aimed at identifying reasons and risk factors associated with the conversion of MAC to GA. These findings may be used to help better anticipate or prevent these events.

## Background

Monitored Anesthesia Care (MAC) is an anesthetic service involving the closely supervised titration of sedatives/analgesics. The American Society of Anesthesiologists (ASA) further defines MAC as an anesthetic service wherein a qualified provider is able to convert to general anesthesia (GA) and rescue a patient’s airway from sedation-induced instability [1]. MAC may be chosen in an effort to reduce post-operative recovery time and/or potential risks of GA [2, 3]. Monitored Anesthesia Care is used in one-third of all ambulatory surgical procedures [4]. Regional anesthesia may also be utilized in combination with MAC to provide better analgesia. However, MAC may prove to be inadequate in some cases and require intraoperative conversion to GA.

Recognizing this risk of conversion from MAC to GA, several studies have identified surgical and patient-related factors associated with this conversion during specific procedures such as transcatheter aortic valve replacements, parathyroidectomies, thyroidectomies, and craniotomies. Reasons for conversion to GA cited in these studies include patient (or surgeon) inability to tolerate MAC, procedural complications, hemodynamic instability, hypoxia, and seizures [5–10]. Additional trials examining the conversion rate of neuraxial anesthesia to GA cited similar reasons [11–13].

Because of the small sample sizes and emphasis on either specific surgical procedures or regional anesthetic techniques, these previous studies are less generalizable to all patients receiving MAC. Given its prevalence of use, the incidence and reasons for “failed MAC” need to be determined for a larger population. The primary goal for the present study was to retrospectively review all non-obstetric MAC cases, with or without regional anesthesia, at a large tertiary academic hospital over the course of more than a decade to determine the overall rate of conversion to GA and to elucidate patient and procedure specific risk factors associated with conversion.

## Methods

The Program for the Protection of Human Subjects/Institutional Review Board at the Icahn School of Medicine at Mount Sinai approved this observational retrospective study and granted a waiver of written consent (IRB # GCO 15–1898). All methods were performed in accordance with the guidelines set forth by the Icahn School of Medicine at Mount Sinai. Obstetric cases were excluded as reasons for conversion from neuraxial to general anesthesia have been studied extensively in prospective cohorts [14].

Our anesthesia information management system (CompuRecord, Philips Medical System, Andover, MA) was queried for all non-obstetric adult patients (> 18 years of age) undergoing MAC and/or MAC with regional anesthesia between July 2002 and July 2015 at the Mount Sinai Hospital in New York City. The primary outcome of this study was evidence of conversion to GA after the start of the surgical procedure. Potential cases of conversion were identified based on documentation in the electronic anesthesia record of insertion of an airway device (endotracheal tube or supraglottic airway), administration of a neuromuscular blocking agent, or free text comments denoting conversion to GA after the start of the surgical procedure. These cases were then reviewed manually to verify conversion. We then reviewed each verified case that was converted to general anesthesia to identify reasons for conversion. These were categorized as either patient-related or procedure-related and then further sub-categorized. In order ensure reliable coding, two authors independently reviewed the cases, and any discrepancies were reviewed and adjudicated by a senior author.

Data were analyzed using SAS 9.4 (SAS inc., Cary, NC, USA). All tests were 2-sided with significance level at 0.05. Descriptive statistics were calculated as N (%) and mean (SD) for the incidence and reasons for conversions as well as for other variables. Continuous variables were compared using the Student’s t-test. Categorical variables were presented as percentages and comparisons were conducted using the Chi-Square test. Multivariate logistical regression using backward elimination was used to identify patient (age, sex, BMI, and ASA-PS), provider (attending alone vs with CRNA/resident), procedure (surgical sub-specialty) factors associated with an increased risk in conversion to GA. All cases with missing data were excluded from statistical analyses.

## Results

Between July 2002 and July 2015, there were a total of 219,061 non-obstetric MAC cases. Of these cases, 3508 were initially identified as MAC cases converted to GA using selected computerized anesthetic events or keywords. After manual anesthetic chart review, we excluded 2385 of these suspected cases as false positives. After exclusion, a total of 1097 MAC cases converted to GA were compiled for analysis, for an overall conversion rate of 0.50% at our institution.

The most common reasons for conversion were failed neuraxial/regional anesthesia (28%), patients’ inability to tolerate MAC (26%), and hypoxia/airway obstruction (14%). Reasons for patients being unable to tolerated MAC included: patient moving around, being disinhibited, and/or complaining of significant pain/discomfort requiring increased levels of analgosedation. Other patient-related complications included hemodynamic instability, aspiration, coughing, adverse reaction to anesthesia, seizures, altered mental status, hypercarbia, poor positioning, and inadequate/loss of intravenous access. Procedural factors, such as change in procedure type, surgeon request, prolonged surgical time, and unpredicted blood loss accounted for 17% of conversions. In 55 cases (5%) the reason for conversion to GA was not documented. (See Table 1).

**Table 1:** Reason for case conversion of monitored anesthesia care (MAC) to general anesthesia (GA)

Sample size, n	1097
Patient-related	
Failed regional anesthesia	303 (27.6)
Patient unable to tolerate MAC	283 (25.8)
Hypoxia/obstruction	150 (13.7)
Aspiration	63 (5.7)
Unknown	54 (4.9)
Hemodynamic instability	38 (3.5)
Other^a^	24 (2.2)
Procedure-related	
Open or another unplanned procedure or surgical extension of area	92 (8.4)
Surgical request	47 (4.3)
Bleeding	22 (2.0)
Extended surgical time	21 (1.9)

^a^Patient coughing, altered mental status, hypercarbia, positioning, adverse event to anesthetic drugs, seizure/seizure-like activity, inadequate IV access

After omitting cases with missing or invalid data, a total of 1052 (96%) of the converted MAC cases and 205,404 of the non-converted MAC cases were included in the univariate analyses. These analyses revealed that when compared with patients in the non-converted group, patients in the converted group had a higher BMI (28.2 ± 6.8 vs. 27.0 ± 6.5; *P* < 0.01). Male patients also had a higher rate of conversion (0.6% vs 0.5%, *P* < 0.001). Cases with a resident or nurse anesthetist included in the care team had a higher rate of conversion when compared to cases where attending anesthesiologists worked alone (0.6% vs 0.2%, *P* < 0.001). Among the surgical specialties, oral maxillofacial surgery (OMFS), otorhinolaryngology (ENT), and orthopedic surgery had the highest rates of conversion. (See Table 2).

**Table 2:** Patient and procedure characteristics of MAC cases converted to GA vs. *not* converted to GA

	MAC cases converted to GA	MAC cases NOT converted to GA	*p*-value^a, b^
Sample size, n	1052	205,404	
Age, mean (SD) in years	57.5 (17.3)	57.8 (17.0)	0.59
Sex, n (%)			< 0.001
Male	556 (0.6)	96,499 (99.4)	
Female	496 (0.5)	108,850 (99.5)	
BMI, mean (SD) in kg/m^2^	28.2 (6.8)	27.0 (6.5)	< 0.001
BMI, n (%)			< 0.001
< 18	35 (0.5)	7104 (99.5)	
18–25	323 (0.4)	83,704 (99.6)	
26–30	325 (0.5)	60,624 (99.5)	
31–35	164 (0.6)	26,577 (99.4)	
> 35	124 (0.7)	18,243 (99.3)	
missing	81 (0.9)	9152 (99.1)	
ASA-PS, n (%)			0.28
1	144 (0.6)	24,924 (99.4)	
2	418 (0.5)	84,879 (99.5)	
3	375 (0.5)	75,951 (99.5)	
4	111 (0.6)	19,040 (99.4)	
5	3 (0.5)	594 (99.5)	
missing	1 (5.9)	16 (94.1)	
Care Team, n (%)			< 0.001
Attending alone	108 (0.2)	46,013 (99.8)	
Resident or CRNA	944 (0.6)	159,391 (99.4)	
Surgical sub-specialities, n (%)			< 0.001
Oral & maxillofacial	8 (2.2)	351 (97.8)	
Otorhinolaryngology	50 (1.4)	3438 (98.6)	
Orthopedics	412 (1.3)	31,136 (98.7)	
Cardiothoracic	3 (0.8)	394 (99.2)	
Gynecology	32 (0.7)	4397 (99.3)	
Vascular	117 (0.5)	21,470 (99.5)	
General	113 (0.5)	21,507 (99.5)	
Interventional Cardiology	44 (0.5)	8625 (99.5)	
Urology	62 (0.5)	11,442 (99.5)	
Unknown	54 (0.4)	15,519 (99.6)	
Gastroenterology	68 (0.2)	37,494 (99.8)	
Other	66 (0.2)	32,833 (99.8)	
Colorectal	8 (0.1)	6097 (99.9)	
Ophthalmology	15 (0.1)	10,701 (99.9)	

Abbreviations: *SD* Standard deviation, *BMI* Body mass index, *ASA-PS* American Society for Anesthesiology Physical Status Classification, *CRNA* Certified registered nurse anesthetist

^a^*p*-value compares MAC cases converted GA and MAC cases not converted to GA

^b^t-test used to compare means and chi-squared test used to compare proportions.

Multivariate logistic regression analyses revealed several patient, provider, and procedure-related risk factors associated with conversion from MAC to GA (Table 3). Age was not independently associated with an increased risk of conversion to GA. Higher BMI was proportionally associated with an increased risk of conversion to GA (BMI > 35, OR: 1.74, 95% CI [1.39–2.17], *P* < 0.0001), as was higher ASA-PS score (ASA-PS 4, OR: 1.64, 95% CI [1.64–2.22], *P* < 0.01). Female patients were less likely to be converted when compared to males (OR: 0.73, 95% CI [0.64–0.84], *P* < 0.0001). Cases involving nurse anesthetists or anesthesia residents were more likely to be converted compared to cases with anesthesiology attendings working independently (OR: 2.03, 95% CI [1.59–2.58]; OR: 1.84, 95% [1.48–2.29], respectively, *P* < 0.0001). Compared with other surgical subspecialties, OMFS, ENT, gynecology, cardiac, general surgery, vascular, and urology were all associated with an increased risk of MAC cases being converted to GA.

**Table 3:** Multivariate logistic regression analyses of risk factors associated with conversion of MAC to GA

Patient and Procedure Characteristics	OR (95% CI)	***p***-value^**a**^
**Age (years)**		
< 35	Reference	
35–65	0.98 (0.79–1.21)	0.84
66–75	0.88 (0.68–1.13)	0.31
76–85	0.94 (0.71–1.24)	0.64
> 85	0.98 (0.67–1.44)	0.93
**BMI (kg/m**^**2**^**)**		
18–25	Reference	
< 18	1.15 (0.78–1.70)	0.48
26–30	1.34 (1.14–1.57)	< 0.001
31–35	1.49 (1.22–1.81)	< 0.001
> 35	1.74 (1.39–2.17)	< 0.001
Missing	1.90 (1.48–2.47)	< 0.001
**Sex**		
Male	Reference	
Female	0.73 (0.64–0.84)	< 0.001
**ASA-PS**		
1	Reference	
2	1.20 (0.97–1.48)	0.09
3	1.36 (1.08–1.72)	0.01
4	1.64 (1.20–2.22)	< 0.001
5	4.04 (0.97–16.85)	0.06
**Care Team**		
Attending	Reference	
Attending with CRNA	2.03 (1.59–2.58)	< 0.001
Attending with resident	1.84 (1.48–2.29)	< 0.001
**Surgical subspecialty**		
Other surgical subspecialties^b^	Reference	
Oral maxillofacial surgery	7.96 (3.74–16.96)	< 0.001
Otorhinolaryngology	6.88 (4.99–9.48)	< 0.001
Gynecology	5.52 (3.73–8.17)	< 0.001
Cardio-thoracic	2.85 (0.90–9.02)	0.07
General surgery	2.71 (2.14–3.42)	< 0.001
Urology	2.50 (1.86–3.37)	< 0.001
Vascular	2.17 (1.69–2.79)	< 0.001

Abbreviations: *OR* Odds-ratio, *CI* Confidence interval, *BMI* Body mass index, *ASA-PS* American Society for Anesthesiology Physical Status Classification, *CRNA* Certified registered nurse anesthetist

^a^*p*-values based on multivariable logistic regression

^b^cardiology, colorectal, gastroenterology, ophthalmology, orthopedics, other, unknown.

Recognizing that failed neuraxial/regional anesthesia was the most common reason for conversion to GA, we ran secondary analyses for the patients that underwent regional anesthesia along with MAC, to determine if any regional anesthetic procedures were associated with an increased risk in conversion to GA. The axillary block was the only regional technique that was found to be significantly associated with increased risk in conversion of MAC to GA (OR: 1.97, 95% CI [1.01–3.85], *P* < 0.05).

## Discussion

In this retrospective review, we identified 219,061 non-obstetric MAC +/− regional anesthesia cases from 2002 to 2015, 1097 (0.50%) of which were converted to GA. This overall rate of conversion was low compared with previous studies, which ranged from 0 to 12%. However, those studies focused on specific surgical procedures (e.g. transcatheter aortic valve replacement, thyroidectomy, parathyroidectomy, and craniotomy) or regional anesthetic techniques (e.g. neuraxial anesthesia) and were limited by smaller sample sizes [5–14]. While our study did not focus on specific surgical procedures, the low conversion rate supports the implementation of MAC for procedures across a number of surgical subspecialties.

With the hope of delineating modifiable risk factors associated with increased rates of conversion, our study identified a myriad of patient, provider, and procedure-specific characteristics associated with an increased risk of conversion to GA. In our study population, obese, male patients were at higher risk for conversion to general anesthesia. The higher risk of conversion found among obese male patients in our study may be explained by the fact that this patient population may also be more likely to have sleep apnea, making them more prone towards hypoxia/airway obstruction - the 3rd most common reason for conversion [15–17]. In particular, a BMI > 35 was the most significant patient-related risk factor associated with conversion to GA.

The increased risk of conversion found among ASA-PS 3 and 4 patients may be due to the lower doses of anesthetic agents that this patient population typically received. Lower doses of anesthetic agents may increase their risk of movement during a MAC case. Alternatively, these sicker patients may be less likely to tolerate adequate doses of anesthesia, succumbing to cardiopulmonary instability leading to conversion to GA.

A variety of different types of surgical procedures were associated with higher rates of conversion to general anesthesia. Orthopedic surgery cases, in particular, were more prevalent in the converted group. This is likely attributable to the increased use of regional/neuraxial techniques in these cases—failure of which was the most common reason for conversion to general anesthesia in our study population. OMFS and ENT cases were also associated with particularly higher odds of conversion to GA. This may be attributable to the notion that procedures in the oral cavity increase the amount of blood and secretions in the oropharynx, which could lead to airway obstruction. Moreover, these procedures carry a higher fire risk than MAC cases in the abdomen or lower body, resulting in lower levels of sedation and possibly decreased patient patient/surgeon tolerability. Several other surgical procedures were associated with higher rates of conversion to GA (e.g. urologic, gynecologic, vascular). Additional studies would be helpful in identifying reasons why these types of procedures have an increased risk of conversion.

Cases with an anesthesia resident or nurse anesthetist in the care team were associated with a higher risk of conversion to GA. It is unknown if this is related to differences in experience/training/skills or to the types of patients/procedures to which these teams were assigned.

We were not able to compare sedative/analgesic medication between the groups because the converted cases did not reliably have a discrete intubation event documented in the electronic records that would allow us to programmatically identify medications given before vs. after conversion to GA. Even if we could do so, this retrospective analysis would not enable us to determine if medications given contributed to the need for conversion or were just an indicator of failing MAC. Finally, the mix of procedure types would confound such a comparison since different procedures require different amounts of analgesia.

The study has several limitations. The data were collected from a single tertiary care hospital in a metropolitan area, potentially limiting the generalizability of these findings. Given the rarity of conversion to GA, some of the predictive variables used in the univariate/multivariate analyses had small sample sizes. As a retrospective review, extracted data quality may have affected the results, and unidentified confounders may influence the observed associations. Procedure-specific detail beyond the level of specialty to find specific at-risk procedures was also beyond the scope and dataset of this study.

## Conclusions

To our knowledge, this is one of the largest studies of intraoperative conversion from MAC to GA. The rates of conversion found here are comparable to smaller studies and reaffirm that MAC is a generally a safe and reliable technique for a large number of procedures, and perhaps should be increasingly utilized for a wide number of procedures. As the practice of monitored anesthesia care continues to expand, it will be important to continue to assess its safety. The patient and surgical-related risk factors associated with conversion to GA identified in this study can be used to help better anticipate or prevent these events.

## Data Availability

The datasets generated and analyzed during the current study are not publicly available due concern for breach of patient confidentiality but are available from the corresponding author on reasonable request.

## Abbreviatons

MAC: Monitored anesthesia care
GA: General anesthesia
BMI: Body mass index
ASA-PS: American Society for Anesthesiology Physical Status Classification
CRNA: Certified registered nurse anesthetist
OMFS: Oral maxillofacial surgery
ENT: Otorhinolaryngology
